# Is diverting loop ileostomy necessary for completion proctectomy with ileal pouch-anal anastomosis? A multicenter randomized trial of the GETAID Chirurgie group (IDEAL trial): rationale and design (NCT03872271)

**DOI:** 10.1186/s12893-019-0657-7

**Published:** 2019-12-12

**Authors:** Laura Beyer-Berjot, Karine Baumstarck, Sandrine Loubière, Eric Vicaut, Stéphane V. Berdah, Stéphane Benoist, Jérémie H. Lefèvre, GETAID Chirurgie group, Y. Panis, L. Maggiori, E. Rullier, Q. Denost, P. Zerbib, E. Cotte, A. Germain, Z. Lakkis, V. Bridoux, J. J. Tuech, M. Ouaissi, G. Meurette, H. Corte, V. Desfourneaux, J. P. Duffas, A. Brouquet, Y. Parc, N. Chafai, C. Debove

**Affiliations:** 1 Department of Digestive Surgery, Hôpital Nord, Assistance Publique-Hôpitaux de Marseille, Aix-Marseille Université, Chemin des Bourrely, 13015 Marseille, France; 2 APHM, Clinic Research Platform, Aix-Marseille Univ, Marseille, France; 3 Department of Clinical Research Fernand Widal Hospital, Assistance Publique-Hôpitaux de Paris, Université Paris VII, Paris, France; 4 Department of Digestive and Oncologic Surgery, Hôpital Bicêtre, APHP, Université Paris Sud, Le Kremlin-Bicêtre, France; 5 Department of Digestive Surgery, AP-HP, Hôpital Saint Antoine, Sorbonne Université, F-75012 Paris, France

**Keywords:** Pouch-anal anastomosis, Ileostomy, Ulcerative colitis, Staged IPAA, Modified 2-stage IPAA

## Abstract

**Background:**

There is no quality evidence of the benefit of defunctioning ileostomy (DI) in ileal pouch-anal anastomoses (IPAAs) performed for inflammatory bowel disease (IBD), but most surgical teams currently resort to DI. In the case of a staged procedure with subtotal colectomy first, completion proctectomy with IPAA is performed for healthy patients, namely, after nutritional support, inflammation reduction and immunosuppressive agent weaning. Therefore, the aim of this trial is to assess the need for systematic DI after completion proctectomy and IPAA for IBD.

**Methods/design:**

This is a multicenter randomized open trial comparing completion proctectomy and IPAA without (experimental) or with (control) DI in patients presenting with ulcerative colitis or indeterminate colitis. Crohn’s disease patients will not be included. The design is a superiority trial. The main objective is to compare the 6-month global postoperative morbidity, encompassing both surgical and medical complications, between the two groups. The morbidity of DI closure will be included, as appropriate. The sample size calculation is based on the hypothesis that the overall 6-month morbidity rate is 30% in the case of no stoma creation (i.e., experimental group) vs. 55% otherwise (control group). With the alpha risk and power are fixed to 0.05 and 0.80, respectively, and considering a dropout rate of 10%, the objective is set to 194 patients. The secondary objectives are to compare both strategies in terms of morbi-mortality at 6 months and functional results as well as quality of life at 12 months, namely, the 6-month major morbidity and unplanned reoperation rates, 6-month anastomotic leakage rate, 6-month mortality, length of hospital stay, 6-month unplanned readmission rate, quality of life assessed 3 and 12 months from continuity restoration (i.e., either IPAA or stoma closure), functional results assessed 3 and 12 months from continuity restoration, 12-month pouch results, 12-month cost-utility analysis, and 12-month global morbidity.

**Discussion:**

The IDEAL trial is a nationwide multicenter study that will help choose the optimal strategy between DI and no ileostomy in completion proctectomy with IPAA for IBD.

**Trial registration:**

ClinicalTrial.gov: NCT03872271, date of registration March 13th, 2019.

## Background

Ulcerative colitis (UC) is a colonic inflammatory bowel disease (IBD) that occurs in young adults. This life-long disease is of public health significance. Indeed, UC prevalence surpasses 0.5% in Europe and is increasing in newly industrialized countries [1]. The proportion of UC patients requiring surgery ranges from 10 to 30% according to the extent of the disease [2, 3]. Restorative proctocolectomy with ileal pouch-anal anastomosis (IPAA) is the gold standard surgery in familial adenomatous polyposis (FAP), UC, and IBD unclassified (IBDU), another colonic IBD formerly called indeterminate colitis [4]. Indeed, this procedure is the only treatment that eliminates both inflammatory and oncologic risks in the colonic and rectal segments. IPAA is a complex procedure, is mostly performed in two or three stages in cases of colonic IBD, and includes a defunctioning ileostomy to avoid anastomotic leakage [4, 5]. In the “traditional” two-stage IPAA, restorative proctocolectomy with IPAA is diverted by an ileostomy, and the second stage is ileostomy closure. In the three-stage IPAA, primary subtotal colectomy (STC) is performed with either end ileostomy or double-end stoma (i.e., ileo-sigmoidostomy). The second stage is the completion of proctectomy with IPAA. The pouch is diverted by a defunctioning ileostomy, which is closed in the third stage 6 to 8 weeks later. In the three-stage IPAA, STC allows the patient to recover from inflammation, malnutrition and immunosuppressive medication. The second stage is usually performed 2 or 3 months later, with the patient in good health. Therefore, the need for a systematic defunctioning ileostomy is questioned, and the “modified” two-stage IPAA has been developed, where STC is followed by completion proctectomy and IPAA, without defunctioning ileostomy.

Indeed, defunctioning ileostomy has several drawbacks, including specific complications (ileostomy prolapse, parastomal hernia, skin erosions), small bowel obstruction (SBO) that occurs in 18 to 22% of the patients [6–8] and dehydration that may require readmission. Defunctioning ileostomy has also associated with negative psychological impacts and a poor quality of life (QoL) before closure [9]. Moreover, stoma closure requires a third surgical procedure under general anesthesia and is associated with a 4% risk for anastomotic leakage. Overall, during the ileostomy period, 8% of patients will require reoperation [10].

An old randomized study from 1992 included 45 patients with no corticosteroid treatment and compared systematic ileostomy during IPAA and no ileostomy. Half of the patients in each group had STC, and the postoperative morbidity was 52% in the “ileostomy” group vs. 32% in the “no ileostomy” group (including an SBO incidence of 22% in the “ileostomy” group vs. 9% in the “no ileostomy” group). These differences were not significant, but the number of patients was small, and these results did not take into account the morbidity of ileostomy closure. Moreover, the mean hospital length of stay (LOS) was significantly longer in the “ileostomy” group than in the in the “no ileostomy” group when both IPAA and ileostomy closure were assessed [6].

More recently, some retrospective case-controlled studies have assessed one-stage IPAA (i.e., total proctocolectomy and IPAA without ileostomy) [11, 12]. The morbidity was comparable between patients who underwent one-stage and those who underwent traditional two-stage IPAA. However, the majority of the patients had FAP, not IBD; all IBD patients were carefully selected, and none received corticosteroid medication. Thus, these results cannot be extended to all colonic IBD patients. Interestingly, a retrospective study emanating from international tertiary care centers found that a “modified” two-stage procedure might be the best option for IBD patients who are at high risk for postoperative morbidity [7]. Namely, Sahami et al. compared 305 IBD patients undergoing IPAA with defunctioning ileostomy to 316 patients undergoing IPAA without defunctioning ileostomy. The rate of anastomotic leakage was not influenced by the ileostomy (ileostomy 16.7% vs. no ileostomy 17.1%, *p* = 0.92), but the ileostomy group presented with more SBO in the long term than the no ileostomy group (ileostomy 18.9% vs. no ileostomy 10.3%, *p* = 0.003). Defunctioning ileostomy was an independent risk factor for SBO (OR = 2.58 CI_95%_[1.5–4.7]). Moreover, the risk of pouch failure over time was similar between the two groups in the Kaplan-Meier analysis.

Two studies compared traditional and modified two-stage IPAA. In 2017, Samples et al. published a comparative nonrandomized prospective study including 248 patients [13]. Despite having patients with more aggressive disease in the modified two-stage group, the 3-year pouch failure rate was similar between the two groups. In their monocenter retrospective study, Zittan et al. included 459 patients [8]. Again, the patients undergoing a modified two-stage IPAA presented with more aggressive disease than those undergoing traditional IPAA, as expected by the indications for primary STC. However, the surgical short-term complications were comparable between the two groups, and the modified two-stage IPAA was an independent protective factor for postoperative fistula (OR = 0.27 IC_95%_[0.12–0.57]) [8].

### Objectives

Based on the abovementioned studies, the overall 6-month morbidity rate may be 55% in cases of ileostomy creation vs. 30% in cases of completion proctectomy with IPAA [6–8, 11–13]. Therefore, we hypothesized that in IBD patients who have had STC, completion proctectomy with IPAA could be performed without defunctioning ileostomy. The aim of the IDEAL trial was to compare IBD patients undergoing completion proctectomy and IPAA with and without defunctioning ileostomy. IDEAL is the French acronym for “diverting loop ileostomy in completion proctectomy with ileal pouch anal-anastomosis”.

## Methods and design

**Fig. 1:**
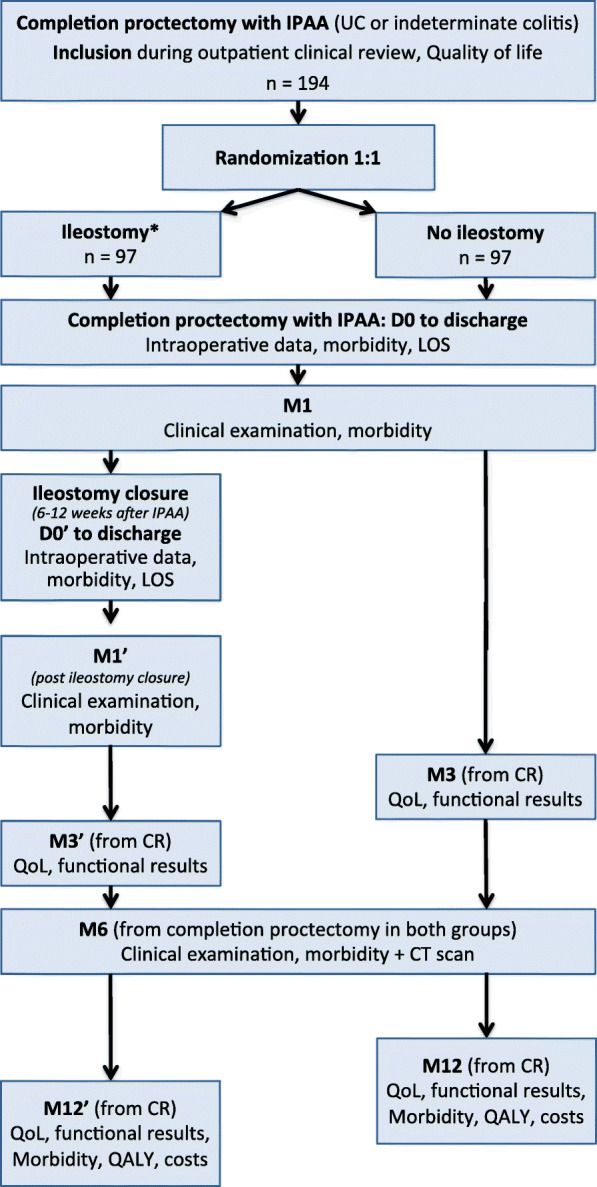
Flow chart of the IDEAL trial *Defunctioning ileostomy, CR: continuity restoration

The IDEAL trial is a nationwide prospective, multicenter, open-label, randomized controlled trial in which IBD patients scheduled for completion proctectomy and IPAA will be randomized between having a defunctioning ileostomy (the ‘ileostomy’ strategy) and not having a defunctioning ileostomy (the ‘no ileostomy’ strategy). The study cannot be blinded because the presence of a stoma cannot be simulated and is obvious to both patients and practitioners. The study duration will be 48 months, and the recruitment duration will be 33 months. The study design is summarized in Fig. 1.

The list of randomization will be established before the implementation of the study with a 1:1 allocation ratio. It will be elaborated under the responsibility of the Clinical Research Platform, Assistance Publique – Hôpitaux de Marseille (APHM). A computer-generated randomized list will be drawn up using a permuted block design. Two stratification indicators will be retained: the center and the body mass index (< or ≥ 25 kg/m^2^).

This trial is supported by a grant from the French Ministry of Health (PHRC-18_0764).

### Endpoints

The primary endpoint is the 6-month global postoperative morbidity, i.e., the number of patients who develop at least one complication at the 6-month follow-up, encompassing both surgical and medical complications, as well as the morbidity of ileostomy closure. The 6-month timepoint is defined from the completion proctectomy and is therefore the same in both groups. We chose a 6-month endpoint because half of the patients will undergo ileostomy closure 6 to 12 weeks after IPAA. Therefore, a one-month or 3-month endpoint would be too short to correctly assess the morbidity of completion proctectomy +/− ileostomy closure. Moreover, a significant part of morbidity related to defunctioning ileostomy occurred in the long-term (i.e., after 30 days) in previously published studies [7, 8].

The secondary endpoints are as follows: 6-month major morbidity based on the Clavien-Dindo classification, [14] i.e., number of patients who develop at least one major complication at the 6-month follow-up; 6-month unplanned reoperation rate, i.e., any unplanned abdominal surgery, whether urgent or elective, other than ileostomy closure; 6-month anastomotic leakage rate assessed by a CT scan with an iodine injection, i.e., pelvic abscess, pelvic extradigestive air, pelvic free fluid or pelvic infiltration; 6-month mortality (linked or not with the disease); initial and cumulative 6-month length of hospital stay; 6-month unplanned readmission rate, i.e., other than for ileostomy closure; global and digestive quality of life (QoL) assessed by the Cleveland Global Quality of Life (CGQL) score [15] and the EuroQol Five-levels (EQ-5D-5 L) (i.e., utility values for health states) [16] at baseline and 3 and 12 months from continuity restoration (i.e., either IPAA or ileostomy closure); functional results assessed by the Cleveland Pouch Functional score (PFS) 3 and 12 months from continuity restoration [15]; 12-month pouch results: pouch failure (i.e., with definite ileostomy), anastomotic stricture, pouch in place, and repeat IPAA 12 months from continuity restoration; 12-month global morbidity (i.e., the number of patients who develop at least one complication 12 months from continuity restoration); and 12-month cost-utility analysis, performed 12 months from continuity restoration (cf. *‘* Economic assessment’ below). Though many redo-IPAAs are performed after one year, it is a secondary endpoint and it was felt not relevant enough in this trial to extend its follow-up.

### Participating centers

The recruitment will be performed in 15 French public academic teaching hospitals. All participating sites will sign a convention with the Direction of Research and Clinical Innovation of APHM for ethical approval before beginning patient inclusion. All these centers are tertiary expert units in IBD, as it has been demonstrated that IPAA should be performed in mid- to high-volume centers, namely, those that perform at least 3 IPAA procedures per year [17].

### Study population

The inclusion and non-inclusion criteria are summarized in Table 1. Completion proctectomy can be indicated after any primary subtotal colectomy, irrespective of both the indication of subtotal colectomy and the time from the previous procedure. UC and IBDU are defined according to the third ECCO consensus guidelines [18]. The exclusion criteria are as follows: wishes to interrupt his/her participation during the study; and no benefits from IPAA (e.g., definite terminal ileostomy). An early stoma closure due to high output with electrolyte imbalances is not an exclusion criterion.

**Table 1:** Inclusion and exclusion criteria

Inclusion criteria	Non-inclusion criteria
– Age ≥ 18 years; – UC or IBDU requiring completion proctectomy with IPAA and who can be treated by both strategies; – Affiliation with or benefitting from a social security system; – Freely signed written informed consent form.	– Indications for total proctocolectomy in a one-stage or traditional two-stage fashion – Crohn’s disease – Pelvic radiotherapy – Indications for total mesorectal excision – Vulnerable patient under the French laws (e.g., minors, pregnant/breastfeeding women) – Participation in another research protocol influencing the postoperative morbidity – No consent to participle, or unable to give a written consent.

The selection criteria are barely restrictive but must be followed to respect the current guidelines. Patients requiring a completion proctectomy after primary subtotal colectomy represent approximately 40% of all colonic IBD patients needing IPAA. It is therefore a frequent situation and restricting the inclusion criteria to completion proctectomy only rather than including all IPAA permits a more homogenous population. Moreover, one-stage IPAA is now restricted to very selected cases of IBD, as explained above in the rationale. The inclusion of patients who present with FAP was estimated to be not relevant, as they scarcely undergo completion proctectomy and do not have rectal inflammation. Select patients who present with colonic Crohn’s disease can undergo IPAA [19]. However, they are not the best candidates for completion proctectomy and IPAA without ileostomy, as they are exposed to a higher risk of pouch failure than UC patients [20]. Therefore, Crohn’s disease patients will not be included in the study.

### Ethics

The research carried out will be in accordance with the Helsinki declaration. This study was submitted to the national drug agency (ANSM) and to the ethics committee “CPP Sud-Est 3” on April 30th, 2019; it was approved after minor corrections. Prior to randomization, written informed consent will be obtained from all patients.

### Study outline

#### Randomization

The investigator will verify the eligibility of the patient with respect to the inclusion and non-inclusion criteria. After the delivery of oral information regarding the study, written consent will be collected (day of inclusion). Both strategies will be explained to the patients before enrollment in the study, along with their pros and cons. For the preregistration evaluation, the included patients will undergo a standard preoperative work-up, as per local protocol. Randomization will be performed by a computer on at least the day before surgery, and the results of the randomization will be given to the patient on at least the day before surgery. All the explanations related to the content of the strategy will again be explained to the patient.

#### Intervention

An anesthesia consultation is planned before the surgery according to the habits of each department. Participation in the study will not alter the anesthetic procedures. The technical modalities of IPAA are not restricted, but it is mandatory that these modalities are identical in both groups for each center in terms of the type of approach (laparoscopic, single-port, robotic), type of anastomosis (hand-sewn or stapled), pelvic drainage and pouch drainage. Open surgery can be the chosen surgical approach for some patients, provided that a mini-invasive approach cannot be performed due to the surgical history or medical contra-indications, as estimated by the surgeon in charge. A J-pouch will be mandatory as it is the pouch of reference [18].

The defunctioning ileostomy is a covering loop ileostomy, which can be fashioned either in the right or left iliac fossa. The use of an ostomy rod is not mandatory and left to the surgeon’s judgment. Ileostomy closure will be performed 6 to 12 weeks after IPAA as per local protocol or later in case of pelvic sepsis. During stoma closure, the type of ileo-ileal anastomosis (whether end-to-end or side-to-side, hand-sewn or stapled) is not restricted. A CT enema scan can be performed before ileostomy closure to assess the absence of anastomotic leakages, as per the local protocol.

The patients randomized in the experimental group will be treated by completion proctectomy and the creation of an IPAA without a defunctioning ileostomy, which corresponds to a modified two-stage IPAA. As defunctioning ileostomy is omitted in this group, an anastomotic leakage test can be performed when deemed necessary by the surgeon in charge. However, the anastomotic leakage test will not be mandatory.

No concomitant care and interventions are prohibited during the trial.

#### Statistical analysis

A sample size calculation will be performed on the hypothesis formulated on the primary endpoint, i.e., the overall morbidity, assessed 6 months post-randomization. Based on previous reports, the overall 6-month morbidity rate is 30% in the case of no ileostomy creation (i.e., experimental group) vs. 55% otherwise (control group) [6–8, 11–13]. With the alpha risk and power fixed to 0.05 and 0.80, respectively, 88 patients need to be included in each group, or 176 in total [Power Analysis and Sample Size Software Version 2008, Utah, USA]. Considering a drop-out rate of 10%, the objective is set to 194 patients.

The full analysis population (including all subjects who will be randomized and will at least be evaluated at baseline) will be used in the primary analysis, and the per-protocol population (including all subjects who will be randomized and will not have major protocol deviations) will be used in the secondary analysis to assess the robustness of the results. The demographic and baseline characteristics will be summarized for the 2 groups (‘control’ and ‘experimental’ groups). The scores of the QoL questionnaires will be calculated from the algorithm provided by the authors of the tools. No comparisons will be provided in accordance with the CONSORT guidelines.

The primary endpoint will be compared between the 2 groups (χ^2^ test or Fisher’s exact test) for the primary analysis. Logistic regression will be performed to adjust for potential confounding factors; variables relevant to the models will be selected based on their clinical interest and/or a threshold *p*-value ≤0.1 during univariate analysis. The final models will express the odd ratios and their 95% confidence intervals. The unadjusted analysis will be the primary analysis, and the adjusted analysis will be a complementary analysis. For the secondary endpoints, quantitative data will be compared using the Student’s t test or Mann-Whitney U test in accordance with the variable distribution, whereas qualitative data will be compared using χ^2^test or Fisher’s exact test, as appropriate.

For the analysis of overall survival, the last date of known contact will be used for subjects who have not died (the subjects will be considered censored). Overall survival will be estimated according to the Kaplan-Meier method. Comparisons between the 2 groups will be performed with the log rank test for the Kaplan-Meier estimates. Cox models will be created.

#### Economic assessment

As recommended by the French High Health Authority (HAS), the clinical endpoint used for the cost-utility analysis will be the QALYs (https://www.has-sante.fr/portail/jcms/r_1499251/en/choices-in-methods-for-economic-evaluation). Preference-based utility scores will be calculated using the EQ-5D-5 L questionnaire. The final result of the economic analysis is the incremental cost-utility ratio (ICUR), which expresses the additional costs per additional QALY. The perspective will be that of the society (HAS recommendation). A second analysis will be conducted from the healthcare provider perspective (i.e.*,* hospital). This analysis will take into account the difference between the costs and the tariffs paid for each cost category. The principal economic analysis will be conducted over the time horizon of the IDEAL trial, i.e., 12-month horizon to capture both the immediate and relevant consequences of both the ‘no ileostomy’ and ‘ileostomy’ strategies, which is a period during which all effectiveness and cost data will be precisely collected. To capture the long-term consequences of the intervention compared, we will extend the time horizon of the cost-utility analysis over the patients’ lifetime. The results of the modeling approach will contribute to the decision-making process by comparing our results with other economic analyses conducted in the field of inflammatory bowel disease and investigating long-term survival and costs.

#### Data collection and monitoring

An electronic Case Report Form (eCRF) will collect the patients’ baseline data (sociodemographics, such as current age, age at UC diagnosis, sex, body mass index, weight gain since subtotal colectomy, ASA score, and smoking status; medical history, such as extent of UC, associated primary sclerosing cholangitis, and associated comorbidities; surgical history, such as indications for primary STC, delay between STC and IPAA, postoperative course of subtotal colectomy, and history of other surgical procedures; current and previous treatments, such as anti-TNF, immunosuppressive treatment, and corticosteroid therapy; and baseline QoL assessment, using CGQL and EQ-5D-5 L) as well as perioperative, short- and long-term data according to the abovementioned endpoints. Each patient will participate in the study for 15 months (as there are 12-month data collect points from continuity restoration, which is performed up to 3 months after IPAA in case of defunctioning ileostomy). Clinical reviews will be performed one and 6 months after IPAA and 3 and 12 months after continuity restoration (i.e., either IPAA or stoma closure).

Monitoring will be conducted to ensure that the data are accurate/complete, the safety and rights of subjects are being protected, and the study is conducted in accordance with the applicable regulatory requirements. No deidentified individual clinical trial participant-level data (IPD) will be shared. This monitoring (including data management and data quality insurance) will be ensured by the sponsor. The following monitoring will be conducted: informed consent, deviation of protocol, data collection, and safety and rights of participants. All the eCRFs will be routed to the coordinating center, APHM (LBB). Data entry will be performed using REDCap. Quality assurance will be performed from the final database (data manager). The database will be forward to the statistical team (Aix-Marseille Univ, APHM, Clinic Research Platform). The study information of this protocol has been posted on clinicaltrials.gov. The status of this trial is pending: the first inclusion is estimated for September 1st^,^ 2019.

#### Patient safety

A safety analysis will be performed by an independent monitoring committee based on the anastomotic leakage rate in the experimental group (i.e., “no ileostomy” group): the chosen threshold will be 15%, as it represents a 30% higher rate of anastomotic leakage compared to the available rates in the literature for cases of completion proctectomy with ileostomy (8 to 16%) [7, 8, 12]. The management of amendments to the protocol and adverse events will follow the procedures of the regulatory department of the sponsor.

#### Dissemination and publication rules

The results of the study will be presented at international, national and departmental meetings and published in an international medical journal under the supervision of the investigator.

## Discussion

Modified two-stage IPAA (i.e., primary subtotal colectomy, then completion proctectomy without defunctioning ileostomy) is a recent and attractive approach in the surgical management of UC, as shown by the growing number of publications in this field since 2016. However, this important topic has never been assessed in a randomized multicenter manner.

The design of the IDEAL trial was discussed and approved by the scientific committee of the *GETAID Chirurgie Group* and will be conducted with its support. This research network includes all French expert surgical centers in inflammatory bowel disease. Beyond this support (hence the participation of all the French expert surgical centers in inflammatory bowel disease), the number of completion proctectomies with IPAA performed over 3 years in France is approximately 423, as shown in a national survey [17]. This is more than twice the number of patients who need to be recruited in the present trial, thus ensuring its feasibility.

Concerns could be raised about the “low threshold” of number of IPAAs per year in the including centers. However, the ECCO guidelines state that high volume centers are needed, but there are no more thresholds in the latest ECCO guidelines, stating “the numbers required to establish a “specialist high volume unit” clearly remain for debate” [21]. Moreover, a French nationwide study [17] has shown that a threshold of 3 IPAAs was significant to reduce mortality. As the study will take place in France, the national authorities have validated this cut-off. However, the including centers are all high volume experts centers in IBD.

Another concern would be the choice of 6-month global morbidity as the primary endpoint. Indeed, patients in the “stoma” group will undergo 2 separate operations, which may be associated to more opportunity for complications than a single operation in the “no stoma” group. However, this has never been demonstrated so far. The morbidity of the modified two-stage IPAA is not well documented and the actual rate of anastomotic leak in the “no stoma” strategy is a major concern. Besides, the 6-month endpoint permits to assess late anastomotic leaks that may be more frequent in the “no stoma” strategy. Finally, specific secondary endpoints such as anastomotic leak or pouch dysfunction will be assessed.

Currently, there is no quality evidence-based medicine concerning the role of the diverting ileostomy for IPAA: the current practice is guided by the data on proctectomy and coloanal anastomosis in rectal cancer. Namely, defunctioning ileostomy reduces the rate of anastomotic leakage and the severity of fistulas after low colorectal or coloanal anastomosis in rectal cancer [22]. Several prospective randomized controlled trials (RCTs) and meta-analyses have confirmed this observation, and thus far, a defunctioning stoma is mandatory after rectal cancer resection with low anastomosis for a usual period of 6 to 8 weeks [23]. Likewise, the majority of IBD patients undergoing completion proctectomy with IPAA also have a temporary defunctioning ileostomy, i.e., undergo a three-stage IPAA, as recommended by the third ECCO consensus [24]. However, patients presenting with UC or IBDU are different from those presenting with rectal cancer: patients with UC or IBDU are usually younger, rarely have a history of pelvic radiotherapy, and usually do not undergo total mesorectal excision.

Except for the underpowered RCT of Grobler et al., only retrospective and nonrandomized studies have assessed defunctioning ileostomy in IPAA, with the inherent risk of multiple biases [7, 8, 13]. Hence, despite encouraging results, the level of evidence is too low to commonly adopt ileostomy omission in IPAA. Moreover, no studies specifically addressed the question of defunctioning ileostomy during completion proctectomy, even though it concerns 36 to 42% of patients undergoing IPAA [7, 17]. The IDEAL study would be the first to assess the role of defunctioning ileostomy during completion proctectomy with IPAA, where the risk of anastomotic leakage is lower and the benefit of defunctioning ileostomy is more questionable. Indeed, completion proctectomy is mostly performed for patients free of corticosteroid medication and in sufficient nutritional status. It would also be the first trial to assess the economic costs of defunctioning ileostomy, as well as its impact on patient QoL. This is of importance as ileostomy creation is always a major fear for patients presenting with UC or IBDU. Finally, balancing patient safety with health-related QoL, hospital LOS and associated costs is critically important for young adults presenting with a life-long colonic affliction.

## Abbreviations

ANSM: National Drug Agency
APHM: Assistance Publique Hôpitaux de Marseille
ASA: American Society of Anesthesiology
CGQL: Cleveland Global Quality of Life
CONSORT: Consolidated Standards Of Reporting Trials
CT: Computed Tomography
DI: Defunctioning Ileostomy
ECCO: European Crohn’s & Colitis Organization
eCRF: Electronic Case Report Form.
EQ-5D-5 L: EuroQol Five-levels
FAP: Familial Adenomatous Polyposis
GETAID: Study Group for the Treatment of Inflammatory Bowel Disease
HAS: French High Health Authority
IBD: Inflammatory Bowel Disease
IBDU: Inflammatory Bowel Disease Unclassified
ICUR: Incremental Cost-utility Ratio
IPAA: Ileal Pouch-Anal Anastomosis
IPD: Individual Participant-level Data
LOS: Length of Stay
PFS: Pouch Functional Score
PHRC: National Clinical Research Program
QALY: Quality Adjusted Life Year
QoL: Quality of Life
RCT: Randomized Controlled Trial
SBO: Small Bowel Obstruction
STC: Subtotal Colectomy
TNF: Tumor Necrosis Factor UC Ulcerative Colitis
